# Altered Caecal Neuroimmune Interactions in the Neuroligin-3^R451C^ Mouse Model of Autism

**DOI:** 10.3389/fncel.2020.00085

**Published:** 2020-04-09

**Authors:** Samiha Sayed Sharna, Gayathri K. Balasuriya, Suzanne Hosie, Jess Nithianantharajah, Ashley E. Franks, Elisa L. Hill-Yardin

**Affiliations:** ^1^School of Health and Biomedical Sciences, RMIT University, Bundoora, VIC, Australia; ^2^Florey Institute of Neurosciences and Mental Health, Parkville, VIC, Australia; ^3^School of Life Sciences, La Trobe University, Bundoora, VIC, Australia

**Keywords:** caecum, mice, autism, neuroimmune, gut-associated lymphoid tissue

## Abstract

The intrinsic nervous system of the gut interacts with the gut-associated lymphoid tissue (GALT) *via* bidirectional neuroimmune interactions. The caecum is an understudied region of the gastrointestinal (GI) tract that houses a large supply of microbes and is involved in generating immune responses. The caecal patch is a lymphoid aggregate located within the caecum that regulates microbial content and immune responses. People with Autism Spectrum Disorder (ASD; autism) experience serious GI dysfunction, including inflammatory disorders, more frequently than the general population. Autism is a highly prevalent neurodevelopmental disorder defined by the presence of repetitive behavior or restricted interests, language impairment, and social deficits. Mutations in genes encoding synaptic adhesion proteins such as the R451C missense mutation in neuroligin-3 (NL3) are associated with autism and impair synaptic transmission. We previously reported that NL3^R451C^ mice, a well-established model of autism, have altered enteric neurons and GI dysfunction; however, whether the autism-associated R451C mutation alters the caecal enteric nervous system and immune function is unknown. We assessed for gross anatomical changes in the caecum and quantified the proportions of caecal submucosal and myenteric neurons in wild-type and NL3^R451C^ mice using immunofluorescence. In the caecal patch, we assessed total cellular density as well as the density and morphology of Iba-1 labeled macrophages to identify whether the R451C mutation affects neuro-immune interactions. NL3^R451C^ mice have significantly reduced caecal weight compared to wild-type mice, irrespective of background strain. Caecal weight is also reduced in mice lacking Neuroligin-3. NL3^R451C^ caecal ganglia contain more neurons overall and increased numbers of Nitric Oxide (NO) producing neurons (labeled by Nitric Oxide Synthase; NOS) per ganglion in both the submucosal and myenteric plexus. Overall caecal patch cell density was unchanged however NL3^R451C^ mice have an increased density of Iba-1 labeled enteric macrophages. Macrophages in NL3^R451C^ were smaller and more spherical in morphology. Here, we identify changes in both the nervous system and immune system caused by an autism-associated mutation in Nlgn3 encoding the postsynaptic cell adhesion protein, Neuroligin-3. These findings provide further insights into the potential modulation of neural and immune pathways.

## Introduction

Emerging evidence suggests that altered communication between the nervous system and inflammatory pathways is associated with multiple diseases including autism. Both altered inflammatory activity (Wei et al., 2011) and a maternal history of autoimmune diseases, such as rheumatoid arthritis and celiac disease, is associated with an increased risk of autism (Atladóttir et al., 2010). The gut-associated lymphoid tissue (GALT) plays a crucial role in mucosal immunity and microbial populations. Caecal patches are lymphoid aggregates located at the blind end of the caecum and contain various immune cells such as macrophages and dendritic cells.

The precise role of the caecum is unclear, but it has been suggested that the appendix in humans houses a “reserve population” of commensal microbes (Randal Bollinger et al., 2007). The caecal patch contributes to gut homeostasis and is a major site for the generation of IgA-secreting cells that subsequently migrate to the large intestine (Masahata et al., 2014). Secretory IgA plays an important role in regulating the activities and compositions of commensal bacteria populations in animal models (Fagarasan et al., 2002; Suzuki et al., 2004; Peterson et al., 2007; Strugnell and Wijburg, 2010). However, whether caecal innervation and immune function are altered in preclinical models of neural disorders is unknown.

Autism is a neurodevelopmental disorder affecting 1 in 59 children (Loomes et al., 2017; Baio et al., 2018). In many autism patients, core features (impairments in social interaction, communication, and repetitive and/or restrictive behaviors) are present along with immunological dysfunction (Marchezan et al., 2018) and gastrointestinal (GI) disorders (Valicenti-McDermott et al., 2006; Buie et al., 2010; Coury et al., 2012). Individuals with autism are four times more likely to experience frequent GI symptoms including alternating diarrhea and constipation, and abdominal pain compared to children with typical development (McElhanon et al., 2014). Interestingly, Inflammatory bowel disease (IBD) is present at significantly higher rates in people with autism than the general public (Kohane et al., 2012). Autism-associated GI dysfunction includes increased GI permeability along with altered motility (Horvath and Perman, 2002; Parracho et al., 2005; Kohane et al., 2012; Neuhaus et al., 2018). Mice expressing the Neuroligin-3 R451C mutation exhibit autism-relevant behaviors including impaired social interaction (Tabuchi et al., 2007; Etherton et al., 2011), a heightened aggression phenotype (Burrows et al., 2015; Hosie et al., 2019), impaired communication (Chadman et al., 2008) and increased repetitive behaviors (Rothwell et al., 2014). Furthermore, the robust aggression phenotype in these mice is rescued by a clinically relevant antipsychotic, risperidone (Burrows et al., 2015), highlighting that this model is useful for preclinical studies. These mice also show altered GI motility, in line with the notion that alterations in the nervous system may also affect the ENS to result in GI dysfunction (Gershon and Ratcliffe, 2004; Hosie et al., 2019).

Most research to date in animal models of autism has focused on replicating the core traits of ASD, in addition to using invasive techniques to highlight changes in neural network activity in the brain (Tabuchi et al., 2007; Halladay et al., 2009; Lonetti et al., 2010; Etherton et al., 2011; Patterson, 2011; Schmeisser et al., 2012; Varghese et al., 2017; Hosie et al., 2018). Using these approaches, it is well established that many gene mutations identified in autism patients affect neuronal function. Here we assessed whether the autism-associated R451C mutation in Neuroligin-3 affects gross caecal morphology, enteric neuronal populations or immune cells within the caecal patch.

## Methodology

### Animals

Adult male NL3^R451C^ mice (8–14 weeks old) and wild type (WT) littermate controls from two different colonies were used in this study. Neuroligin 3 knockout mice (NL3^−/−^; 12 weeks old) were also examined. NL3^R451C^ mutant mice (B6;129-Nlgn3^tm1Sud^/J) were originally obtained from Jackson Laboratories (Bar Harbour, MI, USA) and maintained on a mixed background (mbNL3^R451C^) strain at the Biomedical Sciences Animal Facility, The University of Melbourne (Hosie et al., 2019). These mice were then backcrossed onto a C57BL/6 background for more than 10 generations (i.e., B6NL3^R451C^ mice) and maintained at the animal facility at RMIT University, Bundoora, Australia. In contrast, NL3^−/−^ mice (Radyushkin et al., 2009; Leembruggen et al., 2019) were bred on a C57BL/6NCrl background at the Florey Institute of Neurosciences and Mental Health. All NL3^R451C^ mice were culled by cervical dislocation following RMIT University and The University of Melbourne animal ethics guidelines (AEC# 1727, AEC# 1513519). NL3^−/−^ mice were cervically dislocated and fresh tissue was collected for other applications (AEC# 14095). All data from mutant mice were compared with matched WT littermate controls from the respective cohorts to remove environmental and additional genetic factors (i.e., data from mbNL3^R451C^ animals were compared with mbWT mice; B6NL3^R451C^ vs. B6WT mice and C57BL/6NCrl NL3^−/−^ mice vs. C57BL/6NCrl WT littermates).

All mice from each cohort were housed in mixed genotype groups of up to six per cage to minimize the impact of environmental factors. This study was carried out following the Basel Declaration and all experiments conducted at RMIT University were approved by the RMIT University Animal Ethics Committee and experiments conducted at The University of Melbourne were approved by The University of Melbourne Animal Ethics Committee.

### Caecal Collection

The caecum was collected and weighed from B6NL3^R451C^, mbNL3^R451C^ mice and NL3^−/−^mice. The caecum from each mouse was opened and pinned with the mucosa facing upwards and submerged in 0.1 M PBS on a petri dish lined with sylgard (Sylgard Silicone Elastomer, Krayden Inc., Denver, CO, USA), enabling visualization of the lymphoid patch (i.e., the caecal patch). Images of caecal tissue with a measuring scale were captured and caecal area measured using ImageJ software (ImageJ 1.52a, NIH, Bethesda, MD, USA).

### Wholemount Tissue Preparation

Caecal myenteric and submucosal plexus neurons were revealed by microdissection using fine forceps and dissecting spring scissors. The submucosal plexus was revealed by removing the mucosal layer and carefully exposing neurons adjacent to the circular muscle within the caecal tissue. To obtain the myenteric plexus, the circular muscle was then peeled away from the remaining caecal tissue. A small area of tissue (approximately 0.5 cm^2^) containing myenteric and submucosal plexuses was transferred into a small Petri dish, submerged in 0.1 M PBS for labeling by immunofluorescence.

### Wholemount Immunofluorescence for Neuronal Populations

Immunofluorescence staining was performed on wholemount caecal tissue samples to assess for potential differences in neuronal cell numbers between NL3^R451C^ and WT mice. Wholemount samples of myenteric and submucosal plexus were incubated at room temperature (RT) for 30 min in 0.01% Triton (to permeabilize the tissue for improved access by primary and secondary antibodies) with 10% CAS-block (Invitrogen Australia, Mt-Waverley, Australia; to reduce non-specific binding of antibodies). Then, tissues were incubated with 30 μl primary antisera; human anti-Hu (1:5,000, a pan-neuronal marker; a gift from Dr. V. Lennon, Mayo Clinic, Rochester, MN, USA) and sheep anti-neuronal Nitric Oxide Synthase (NOS; 1:400; Abcam, Eugene, OR, USA) and kept at 4°C overnight in a sealed container. After incubation, caecal tissues were washed with 0.1 M PBS (three washes of 10 min duration). Secondary antisera (30 μl) were applied to the samples and left for 2.5 h at RT on a shaker incubator (Digital Shaking Incubator OM11, Ratek, Australia). Caecal tissues were mounted using fluorescence mounting medium (DAKO Australia Private Limited; Botany, NSW, Australia).

### Imaging of Caecal Neuronal Populations

Images of caecal tissue containing the submucosal, myenteric plexus were analyzed using ImageJ (ImageJ 1.52a, NIH, Bethesda, MD, USA) and Imaris software (Imaris 64X 9.1.0; Bitplane AG, UK). 10 myenteric ganglia and 10 submucosal ganglia were selected from each wholemount caecal tissue sample (*n* = 5 NL3^R451C^ and *n* = 5 WT samples). From each ganglion, the number of Hu and NOS stained cells were counted.

### Caecal Patch Tissue Collection

Caecal tissues including caecal patch samples were fixed in 4% formaldehyde solution at 4°C overnight. The next day, tissue samples were washed three times (10 min per wash) with filtered 0.1 M PBS. The caecal patch was excised from the caecal tissue using spring scissors. Caecal patch samples were subsequently placed into a 30% sucrose solution in distilled water overnight at 4°C for cryoprotection. Caecal patches were placed in a cryomold (Tissue-Tek Cryomold, Sakura, Finetek, USA) filled with optimal cutting temperature compound (Tissue-Tek, OCT compound, Sakura, Finetek, USA). Cryomolds containing caecal patch samples were then snap frozen using liquid nitrogen and tissue blocks stored at −80°C. Frozen caecal patch samples were sectioned at 6-micron thickness using a cryostat (Leica CM1950 Clinical Cryostat, Leica Biosystems Nussloch GmbH, Germany) and collected on positively charged slides (Thermo Fisher Scientific, Waltham, MA, USA Menzel-Glaser, Superfrost^R^ plus, New Hampshire, USA and stained for Haematoxylin & Eosin (H&E) to assess for overall cell density.

### Caecal Patch Image Analysis

Images were obtained using an Olympus slide scanner microscope (VS120-S5; Olympus Australia Private Limited; Melbourne, VIC, Australia) and the cell density within the caecal patch was analyzed using ImageJ software (ImageJ v1.52a, NIH, Bethesda, MD, USA). The entire area of each caecal patch was selected to calculate the area of the caecal patch and cell numbers within that area. The total number of cells was then divided by the area of interest to calculate the number of cells per 100 μm^2^.

### Caecal Patch Immunofluorescence

Immunofluorescence was also performed on cross-sections of caecal patch tissue samples to assess for altered density and morphology of macrophages. To observe a subpopulation of immune cells within the caecal patch, immunofluorescence for the immune cell marker Iba-1 (1:3,000, Abcam, USA) was conducted. The sections were incubated for 30 min with 0.1% triton and 10% CAS-block at RT. Thirty microliters of primary antibody was subsequently applied to each section and kept at 4°C overnight in a moisture sealed container. After incubation, caecal patch sections were washed with 0.1 M PBS (3 × 10 min washes). Secondary antiserum was applied to the samples and left for 2.5 h at RT on a shaker incubator. Caecal sections were mounted using fluorescence mounting medium (DAKO Australia Private Limited; Botany, Australia) containing DAPI (4′,6-diamidino-2-phenylindole) and stored at 4°C overnight. Tissue samples were imaged using a confocal electron microscope (Nikon Confocal Microscope: A1; Version 4.10). A Z-series of images of caecal patch sections (30 μm thickness) were captured and saved in the ND2 file format. Imaris software (Imaris 64X 9.1.0; Bitplane AG, UK) was used for 3D cellular reconstruction of Iba-1 labeled macrophages.

### Statistical Analysis

Potential statistical differences between groups were identified using Student’s *t*-tests.

## Results

Mouse body weight, caecal weight, and caecal tissue area were assessed to determine if anatomical changes occur in the presence of the autism-associated R451C mutation in mice. To address whether the R451C mutation and the *Nlgn3* gene itself plays a broader role in caecal weight, ceacae from NL3^R451C^ mice bred on two different background strains were weighed, and caecal weights from mice lacking Nlgn3 compared to WT littermates were also compared.

**Figure 1:**
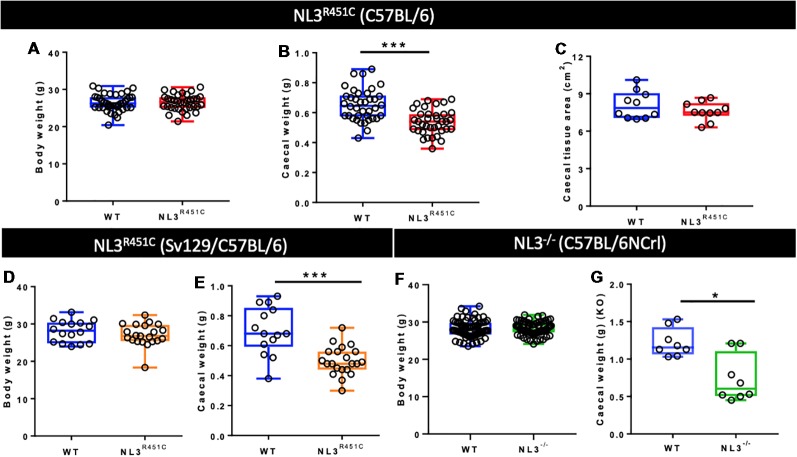
Bodyweight, caecal weight, and caecal tissue area. **(A)** Pure C57BL/6 background wild type (WT) and NL3^R451C^ mice (red) show similar body weights. WT (*n* = 39) and NL3^R451C^ (*n* = 34) mice; *p* = 0.88. **(B)** Caecal weight is reduced in NL3^R451C^ C57BL/6 background mice; WT (*n* = 38) and NL3^R451C^ (*n* = 36) mice. **(C)** Similar caecal tissue area for WT (*n* = 15) and NL3^R451C^ (*n* = 16) mice. **(D)** In mixed background mice (orange), no differences in body weight were found. WT (*n* = 16) and NL3^R451C^ (*n* = 21) mice; *p* = 0.30. **(E)** Caecal weight is also reduced in NL3^R451C^ (orange) mixed background mice. WT (*n* = 14) and NL3^R451C^ (*n* = 21) mice). **(F)** Body weight is unchanged in a large cohort of WT (*n* = 70) and NL3^−/−^ (*n* = 71) mice; *p* = 0.95. **(G)** Reduced caecal weight in NL3^−/−^ (green) mice. WT (*n* = 8) and NL3^−/−^ (*n* = 8) mice. Student’s *t*-test *p < 0.05; ***p < 0.001. Each symbol indicates an individual mouse. Mixed background mice were bred on a mixed Sv129/C57BL/6 genetic background; KO: NL3^−/−^ mice (bred on C57BL/6NCrl mice).

The average body weight of WT (*n* = 39) and NL3^R451C^ (*n* = 34) mice was similar (26.38 ± 0.4 g and 26.46 ± 0.4 g, WT and NL3^R451C^ respectively; *p* = 0.88; Figure 1A). To determine if the reduction in NL3^R451C^ caecal weight was due to a reduction in the size of the caecum itself, total caecal tissue area was measured. No difference between the caecal area of WT (*n* = 15) and NL3^R451C^ (*n* = 16) mice was observed (7.99 ± 0.36 and 7.75 ± 0.5 cm^2^, respectively; *p* = 0.51; Figure 1B). To determine if the R451C mutation affects caecal structure in mice, the fresh caecal weight from 38 WT and 36 NL3^R451C^ mice was recorded. NL3^R451C^ caecae were significantly lighter than WT (0.65 ± 0.02 g and 0.54 ± 0.01 g, WT and NL3^R451C^ respectively; *p* = 0.0001; Figure 1C). A role for the *Nlgn3* gene in influencing caecal weight is supported by similar observations in NL3^R451C^ mice bred on a mixed background strain and in Nlgn3^−/−^ (NL3^−/−^) mice in which the *Nlgn3* gene is deleted. In mice expressing the R451C mutation bred on a mixed background (mb) strain, the average body weight was similar (28.11 ± 1.01 g and 27.1 ± 0.9 g, WT and mbNL3^R451C^ *n* = 16 and *n* = 21, respectively; *p* = 0.30; Figure 1D). Caecal weight was also reduced in mb strain mutant littermates (0.69 ± 0.11 g, 0.49 ± 0.28 g; WT (*n* = 14) and mbNL3^R451C^ (*n* = 21), respectively; *p* < 0.0001; Figure 1E). Bodyweight is unchanged in a large cohort of WT (*n* = 70) and NL3^−/−^ (*n* = 71) mice aged 10–12 weeks; *p* = 0.95; Figure 1F. Similar to data from both the C57BL/6 and mb strains of NL3^R451C^ mice, KO (NL3^−/−^) mice also revealed a reduction in caecal weight (1.16 ± 0.5 g and 0.61 ± 0.53 g; WT and NL3^−/−^, respectively, *n* = 8 in each group; *p* = 0.02; Figure 1G). These findings suggest a role for the *Nlgn3* gene in regulating caecal weight in mice.

**Figure 2:**
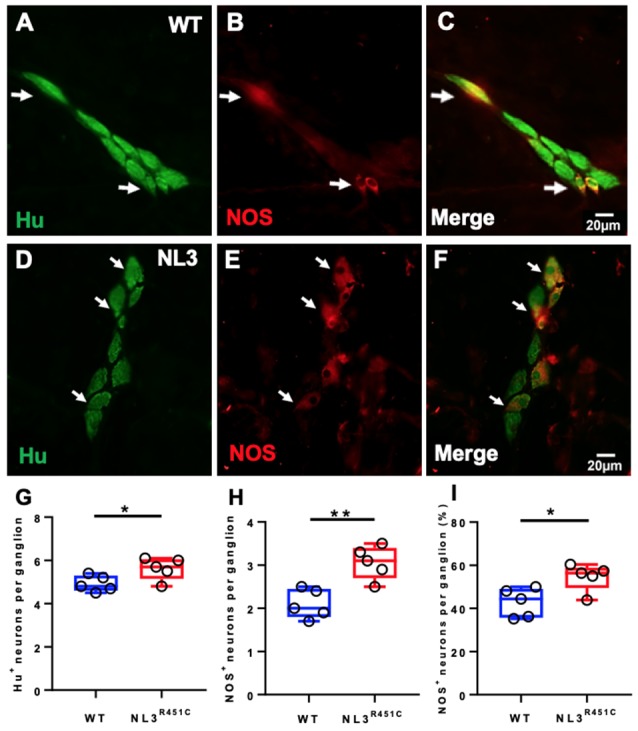
Caecal submucosal neuronal numbers and proportions. WT caecal submucosal plexus ganglia labeled for Hu **(A)** Nitric Oxide Synthase (NOS; **B**); and overlap illustrated in the merged image **(C)**. NL3^R451C^ caecal submucosal plexus ganglia labeled for Hu **(D)** NOS **(E)**; merge **(F)**. Scale bar = 20 μm. **(G)** The total number of Hu labeled neurons per ganglion. **(H)** The total number of NOS stained cells per ganglion. **(I)** Proportions of NOS stained neurons/ganglion; *n* = 5 in each group. Each symbol indicates an individual mouse. Bars in boxplots indicate the mean and range of the data. White arrows indicate neurons immunoreactive to both Hu and NOS. Student’s *t*-test. *P < 0.05; **P < 0.01.

To investigate whether the NL3^R451C^ mutation alters neural populations in the caecal submucosal and myenteric plexus, immunofluorescence for the pan-neuronal marker Hu and NOS (which labels approximately 20–40% of myenteric neurons capable of synthesizing NO (Sang and Young, 1996), the major inhibitory enteric neurotransmitter of the ENS) was conducted. Wholemount preparations of WT (Figures 2A–C) and NL3^R451C^ (Figures 2D–F) submucosal plexus were labeled with Hu and NOS to quantify neuronal subpopulations. The total number of neurons (i.e., labeled by Hu) per submucosal ganglion was increased in NL3^R451C^ mice (5 ± 0.2 and 6 ± 0.2 neurons, WT and NL3^R451C^, respectively, *n* = 5 in each group; *p* = 0.04; Figure 2G). Similarly, NL3^R451C^ mice showed increased numbers of NOS immunoreactive neurons per ganglion (2 ± 0.2 and 3 ± 0.2 cells; WT and NL3^R451C^ respectively, *n* = 5 in each group; *p* = 0.003; Figure 2H). In submucosal neurons, there was also an increased percentage of NOS neurons per ganglion in WT and NL3^R451C^ mice (43 ± 3% and 55 ± 3%; WT and NL3^R451C^ respectively; *p* = 0.02; Figure 2I).

**Figure 3:**
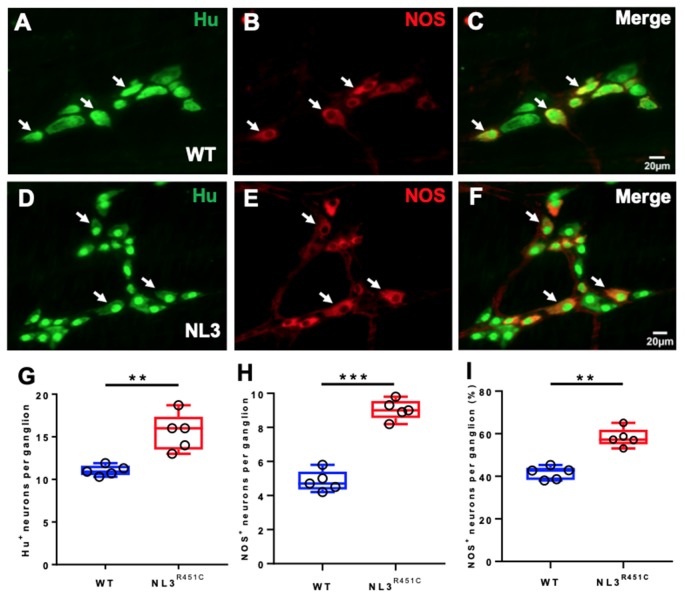
Caecal myenteric neuronal numbers and proportions. WT caecal submucosal plexus ganglia labeled for **(A)** Hu (green), **(B)** NOS (red) and **(C)** merge. NL3^R451C^ caecal submucosal plexus ganglia labeled for **(D)** Hu (green) **(E)** NOS (red), **(F)** merge. Scale bar = 20 μm. **(G)** The number of Hu^+^ neurons/ganglion. **(H)** The number of NOS immunoreactive neurons/ganglion. **(I)** The percentage of NOS neurons/ganglion; *n* = 5 in each group. Each symbol indicates an individual mouse. Bars in boxplots indicate the means and range of the data. White arrows indicate neurons immunoreactive to both Hu and NOS. Student’s *t*-test **p < 0.01; ***p < 0.001.

Wholemount preparations of WT (Figures 3A–C) and NL3^R451C^ (Figures 3D–F) myenteric plexus were labeled with Hu and NOS. Similar to findings in the submucosal plexus, more myenteric neurons (labeled for Hu) were seen in NL3^R451C^ mice (11 ± 0.3 and 15 ± 1 neurons/ganglion, WT and NL3^R451C^ respectively, *n* = 5 in each group; *p* = 0.002; Figure 3G). The number of NOS stained caecal myenteric neurons per ganglion was also increased in NL3^R451C^ mice (5 ± 0.3 and 9 ± 0.2 neurons/ganglion, WT and NL3^R451C^, respectively, *n* = 5 in each group; *p* < 0.0001; Figure 3H). The percentage of NOS stained neurons per myenteric ganglion was also increased in NL3^R451C^ mice (41 ± 1.3% and 58 ± 2.0%; WT and NL3^R451C^ respectively; *p* = 0.0001; Figure 3I). These data show that the R451C mutation results in increased numbers of caecal submucosal and myenteric neurons in mice.

**Figure 4:**
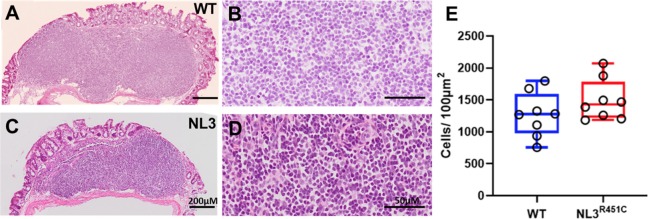
Caecal patch cell density. Haematoxylin and Eosin (H&E) stained transverse sections of caecal patches from WT **(A,B)** and NL3^R451C^ **(C,D)** mice. **(E)** There was no difference in overall caecal patch cell density in WT (*n* = 8) and NL3^R451C^ mice (*n* = 8). Each symbol indicates an individual mouse. Bars in boxplots indicate the mean and range of the data.

To assess whether the R451C mutation alters the GALT structure, we measured total cell density in H&E stained cross-sections of the caecal patch of WT (Figures 4A,B) and NL3^R451C^ (Figures 4C,D) mice. Caecal patch cellular density was similar in both genotypes (1276 ± 48 and 1428 ± 22 cells/100 μm^2^, WT and NL3^R451C^ mice respectively; *n* = 8 in each group; *p* = 0.28; Figure 4E).

**Figure 5:**
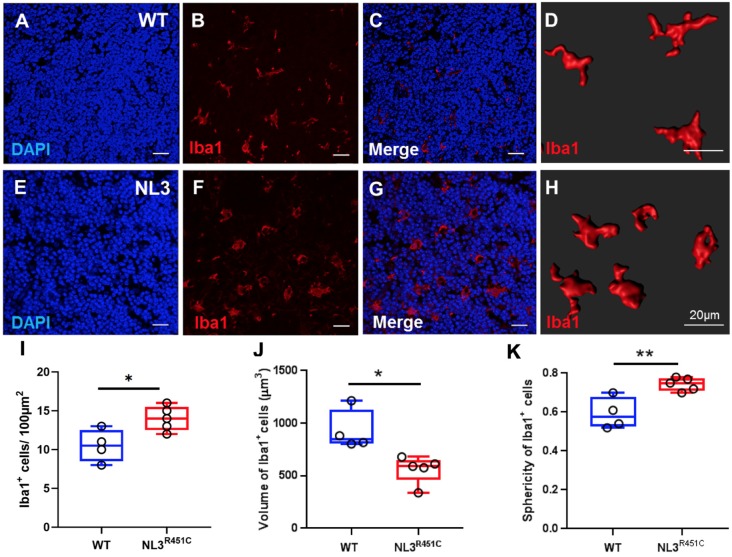
Caecal patch macrophage density and morphology. WT caecal patch tissue labeled for **(A)** DAPI and **(B)** Iba-1, **(C)** merge; **(D)** 3-D reconstruction of Iba-1 labeled cell morphology. NL3^R451C^ caecal patch tissue labeled for **(E)** DAPI **(F)** Iba-1, **(G)** merge, **(H)** 3-D reconstruction of Iba-1 labeled cell morphology. **(I)** Density of Iba-1 stained cells in WT and NL3^R451C^ caecal patch tissue. **(J)** Volume of Iba-1 stained cells in WT and NL3^R451C^ caecal patch tissue. **(K)** Sphericity of Iba-1 stained cells in WT and NL3^R451C^ mice. Each symbol indicates an individual mouse (*n* = 4 WT and *n* = 5 NL3^ R451C^). Bars in boxplots indicate the mean and range of the data. Student’s *t*-test *p < 0.05; **p < 0.01. Scale bars = 20 μm.

In healthy intestinal mucosa, mononuclear phagocytes comprising both macrophages and dendritic cells are the most abundant leukocyte population and play an important role in maintaining homeostasis (Kühl et al., 2015). However, little is known about the morphology and role of macrophages associated with GALT in the intestine (den Haan and Martinez-Pomares, 2013) such as the caecal patch. Caecal patch samples were labeled with the pan-nuclear marker, DAPI, and a pan-macrophage antiserum targeting the ionized calcium-binding adaptor molecule 1 (Iba-1) to determine whether the R451C mutation affects these immune cells in WT (Figures 5A–D) and NL3^R451C^ (Figures 5E–H) within the caecal patch. NL3^R451C^ caecal patch tissue had a higher density of Iba-1 stained cells (14 ± 0.7 cells/100 μm^2^, *n* = 5) compared to WT mice (10.5 ± 1 cells/100 μm^2^, *n* = 4; *p* = 0.02; Figure 5I). The volume of Iba-1 stained cells in WT was larger than in NL3^R451C^ mice (928.5 ± 97 μm^3^ and 559.7 ± 58 μm^3^; WT (*n* = 4) and NL3^R451C^ (*n* = 5), respectively; *p* = 0.01; Figure 5J). Iba-1 stained cells in NL3^R451C^ mice showed increased sphericity (0.6 ± 0.04 and 0.7 ± 0.02 arbitrary units; WT (*n* = 4) and NL3^R451C^ (*n* = 5) respectively; *p* = 0.007; Figure 5K). These results suggest that the autism-associated R451C mutation in *Nlgn3* alters macrophage density and morphology within the caecal GALT.

## Discussion

The nervous system and the immune system are in constant bidirectional communication (reviewed in Margolis et al., 2016). Altered immune responses and gut dysfunction commonly occur in individuals genetically susceptible to autism (Coury et al., 2012). Altered neuronal communication in autism (Betancur et al., 2009; Grabrucker et al., 2011; Huguet et al., 2016), likely contributes to changes in the peripheral nervous system, and therefore GI function (Hosie et al., 2019; Leembruggen et al., 2019).

A main finding from this study is the clear reduction of caecal weight in mice expressing the Neuroligin-3 R451C mutation. Importantly, in addition to our findings on a pure C57BL/6 genetic background, caecal weight was also reduced in mice bred on a mixed background in a different animal facility. These findings, therefore, confirm a persistent effect of the gene mutation and rule out genetic susceptibility due to background strain or environment. Furthermore, mice lacking Neuroligin-3 expression (NL3^−/−^ mice) that were bred in a third animal facility, and therefore experienced a different environment to the two NL3^R451C^ strains, also had reduced caecal weight. Together, these findings suggest that the Nlgn gene plays a role in caecal neuroimmune physiology and that the reduction in weight is unlikely solely due to diet, microbial populations, and other environmental factors. A reduction in caecal weight has also been reported in a mouse model of obesity. For example, obese mice fed a high-fat diet (diet-induced obese mice) had caecal weights approximately 50% reduced compared to controls, and this reduction was restored by antibiotic treatment (Soto et al., 2018). Since obesity is associated with increased inflammation, our observations in NL3^R451C^ mice might also indicate elevated inflammatory cytokine levels, which remain to be assessed.

The reduced caecal weight in NL3^R451C^ mice may indicate changes in caecal mucus thickness. The hydrophilic mucus layer that coats the GI tract plays an important role in innate host defense (Mowat, 2003). Changes in the mucus thickness could contribute to an altered immune response in the host organism (Liévin-Le Moal and Servin, 2006; McGuckin et al., 2009). Accordingly, altered mucus thickness along the GI tract may contribute to GI dysfunction which is commonly observed in children with autism. Based on studies in preclinical models of other disorders, aberrant mucus production may be present alongside other phenotypic traits. For example, caecal tissue sampled from a mouse model of stroke (72 h after brain injury) showed decreased numbers of mucus-producing goblet cells compared to sham-treated mice (Houlden et al., 2016). Reductions in goblet cell number and size were also reported in mice during the development of ulcerative colitis (Van der Sluis et al., 2006; Johansson et al., 2008). Although potential changes in caecal weight were not correlated with these observations, a thinning of the adherent mucus layer and reduced total mucus volume within the caecum may contribute to the significant reduction in caecal weight in NL3^R451C^ mutant mouse strains identified here.

The enteric nervous system (ENS) regulates GI motility and secretion, as well as nutrient uptake and gut immune and inflammatory processes (Goyal and Hirano, 1996). The two main cell populations of the ENS are neurons and enteric glial cells (EGCs; Jessen, 2004). Many studies have identified enteric neuron pathologies in the context of inflammatory disease (Marlow and Blennerhassett, 2006; Boyer et al., 2007; Winston et al., 2013; Talapka et al., 2014; Rahman et al., 2015; Li et al., 2016), but how alterations in the ENS might affect inflammatory pathways remains largely unknown. Nevertheless, altered neuronal activity has previously been implicated in altering immune function, where reports investigating NO levels in human colonic and rectal mucosal biopsies in active ulcerative and Crohn’s disease showed elevated expression of Nitric oxide synthase (NOS; Rachmilewitz et al., 1995; Ljung et al., 2006).

Changes in enteric neuronal numbers are reported in animal models demonstrating GI dysfunction (Schneider et al., 2001; de Fontgalland et al., 2014; Hosie et al., 2019). Our findings that both submucosal and myenteric neuronal numbers are increased in NL3^R451C^ mice caecal tissue indicate that the R451C mutation likely alters neuronal populations during development. These results are in agreement with our previous report showing increased jejunal neuronal numbers in adult NL3^R451C^ mice bred on a mixed genetic background (Hosie et al., 2019). In addition to a potential developmental effect, these findings suggest that the NL3^R451C^ mutation may influence caecal function. Specifically, we speculate that the R451C mutation could alter the rhythmic caecal “churning” of waste that occurs post digestion and before expulsion *via* the colon, however, this hypothesis remains to be investigated. The contractile activity of the GI tract is neurally regulated so given that the R451C mutation is expressed in the gut in these models (Hosie et al., 2019), it would indeed be of interest to assess whether NL3^R451C^ mice show altered caecal motility.

In addition to characterizing changes in enteric neuronal populations in NL3^R451C^ mice, we investigated the effects of the autism-associated R451C mutation on macrophages in caecal tissue using the pan-macrophage marker, Iba-1. NL3^R451C^ mice showed increased numbers of Iba1 stained cells in caecal patch tissue compared to WT mice. Also, the volume of Iba-1 immunoreactive cells was decreased and are more spherical in NL3^R451C^ mutant mice compared to WT littermates. These findings could indicate that macrophages within NL3^R451C^ caecal patch tissue are present in a more reactive state compared to WT mice, with potential implications for immune pathways in this model. Similar observations were reported in disease conditions such as IBD, where both the number and morphology of intestinal macrophages are altered (Mowat and Bain, 2011; Bain and Mowat, 2014). Moreover, macrophages are integral to the pathogenesis of Crohn’s disease (Smith et al., 2011).

In summary, to further assess the impact of the neuroligin-3 R451C mutation on both the enteric nervous system and the immune responses within the caecum, experiments should investigate changes in mucus properties, potential alterations in the mucus-producing goblet cells of the epithelium, inflammatory pathways, and caecal function; including permeability and motility, in this model. Each of these areas of investigation will yield valuable findings about the fundamental role of the caecum in mice as well as the pathophysiology resulting from this mutation.

## Conclusion

This is the first study to assess the impact of the Neuroligin-3 R451C mutation on caecal structure at both an anatomical and cellular level in mice. The observation that the Neuroligin-3 gene plays a role in regulating caecal weight across multiple genetic backgrounds and environments identifies a new role for the *Nlgn3* gene in mice. This work also highlights the caecum as a region of interest within the GI tract that may play a central role in modulating neuro-immune interactions. In the context of neurodevelopmental disorders, our findings that an autism-associated mutation that affects nervous system function also impacts GALT have implications for identifying novel interactions between the enteric nervous system, microbes located within the gut lumen, immune pathways and potential therapeutic targets for GI dysfunction.

## Data Availability Statement

The datasets generated for this study are available on request to the corresponding author.

## Ethics Statement

The animal study was reviewed and approved by RMIT University and The University of Melbourne animal ethics committees (AEC# 1727, AEC#1513519).

## Author Contributions

GB, SH, AF, and EH-Y conceptualized, supervised and designed the research. SS, EH-Y, GB, and JN harvested the tissue and SS and GB undertook the research. SS, SH, and EH-Y drafted the initial version of the manuscript. All authors read and contributed to the final manuscript.

## Conflict of Interest

The authors declare that the research was conducted in the absence of any commercial or financial relationships that could be construed as a potential conflict of interest.
